# 

**Published:** 1848-10

**Authors:** 


### GENERAL INDEX FOR FIRST VOL.

Owing to indisposition, we felt it our duty to leave the city before the last No. of the 1st Vol. was through the press—and to this cause may be attributed also the oversight in this particular. We therefore send this No. of the Register to all who subscribed for the 1st Vol., and enclose therewith an index.

We are now happy to say that our health is perfectly restored, and if the 2d Vol. of the Register does not present an improved appearance it shall not be our fault. It is now to be printed on better paper, with new type, and will be, when necessary, illustrated with suitable engravings. The cost as a matter of course, is considerably increased—hence, if the Profession wish to see the work sustained, they must send in their subscriptions—to pay is *actually* to subscribe.—Cin. Ed.

### TO OUR CORRESPONDENTS.

A case of Hæmorrhage, by Dr. J. A. W., will appear in our next. Also, an interesting letter from Dr. B. T. W., of Tenn. We hope both these gentlemen will favor us again.

Several selections and editorial notices sent in by the Louisville and St. Louis Editors, are crowded out of this No.

The Necessity of a Correct Professional Education, by Dr. K., will also appear in our next.—Cin. Ed.

### TO SURSCRIBERS.

It will be recollected that the terms of the Register, are two dollars, payable in advance. We shall continue to send the Register, after the present number, to such only as comply with these terms. We hope therefore, that those who intend taking the second volume, and desire to sustain such a work, will send in their subscriptions promptly.—CIN. ED.

---

### AMERICAN JOURNAL AND LIBRARY OF DENTAL SCIENCE.

The September No. of this valuable Journal has not yet come to hand. We can appreciate all the unavoidable delays in the regular issuing of such a work, yet, we hope soon to see this again on our table.—CIN. ED.

